# Advanced Age, Female Gender and Delay in Pacemaker Implantation May Cause TdP in Patients With Complete Atrioventricular Block

**Published:** 2010-10-31

**Authors:** Omer Yiginer, Fethi Kilicaslan, Mustafa Aparci, Zafer Isilak, Omer UZ, Fatih Bayrak, Elif Eroglu, Mehmet Uzun, Ejder Kardesoglu, Ata Kirilmaz, Bekir Sitki Cebeci

**Affiliations:** 1 GMMA, Haydarpasa Training Hospital, Cardiology Division, Istanbul-TURKEY; 2 Yeditepe University, School of Medicine, Cardiology Division, Istanbul -TURKEY

**Keywords:** Long QT, complete AV block, torsades

## Abstract

**Aim:**

We aimed to report the clinical features related to torsades de pointes (TdP) in patients with complete AV block (CAVB).

**Methods:**

Patients with CAVB who were admitted to our instituition between January 2007 and January 2010 were retrospectively evaluated in terms of the occurence of TdP. The clinical features were compared in patients with and without TdP using the software of SPSS.

**Results:**

Sixty-four patients were determined to have CAVB. Three of them had documented episodes of TdP. All three patients experiencing TdP were females, whereas 48% of the patients with CAVB were females. The mean age of patients with TdP was significantly greater than the mean age of the other patients (85 ±3 vs. 78±7.6, respectively; p<0.05). In our archives, bradycardia exposure time could be determined in only 49 patients without TdP. Among them, just 10 patients had been exposed to bradycardia over 48 hours, whereas all of the 3 patients with TdP had been exposed to bradycardia over 48 hours (p<0.05). Additionally, in two patients with TdP, we demonstrated  that QT and QTc prolongation increases as the duration of bradycardia is extended. Furthermore, all patients with TdP had notched T waves in the ECG on the occurrence day of TdP, whereas they did not have any notched T wave in their ECG on the admission day.

**Conclusion:**

Among the patients with CAVB, elderly females are more susceptible to development of TdP. Delay in institution of physiological heart rate leads to further QT prolongation and thereby to TdP. Besides QT prolongation, the finding of T wave notching on ECG may also have a predictive value for TdP.

## Introduction

Complete atrioventricular block (CAVB) is potentially lethal and therefore, an emergent clinical condition. Impulses generated in the atria can not propagate to the ventricles due to a defect in the atrioventricular node and/or its adjacent conductive tissue. Temporary transvenous pacemaker (PM) implantation and transthoracic pacing are lifesaving treatment methods which could be performed at either bedside or in laboratory. Decision of PM implantation is particularly determined by the presence or absence of symptoms directly attributable to bradycardia. However, CAVB may potentially be associated with lethal ventricular arrhytmia. CAVB may predispose to acquired long-QT syndrome (LQTS) and torsade de pointes (TdP) [1,2].  The QT interval is the electrocardiographic manifestation of ventricular depolarization and repolarization. Prolonged QT interval and repolarization may cause early after depolarizations (EADs). EADs that reach the threshold voltage cause ventricular extrasystoles and tachycardias. In fact, Dessertenne had described the first TdP in a patient with CAVB [3]. In terms of patient management, it is essential to determine patients who are susceptible for the development of torsades de pointes. In this article, we aimed to evaluate the clinical and electrocardiographic features foretelling TdP in CAVB.

## Methods

All patientsts with CAVB who were admitted to our department between January 2007 and January 2010 were enrolled for the study. TdP was defined as a ventricular tachycardia (faster than 150 beats/min and lasting  5 beats) with a polymorphic configuration. Patients with CAVB occurring during acute myocardial infarction or vasovagal syncope were excluded. Data are presented as mean ± SD. The variables were compared between groups using Chi-square test or Mann-Whitney U tests, as appropriate. The p values < 0.05 were considered statistically significant.

## Result

Sixty-four patients were determined as having CAVB. Three of them had documented episodes of TdP. Clinical features of the patients with and without TdP are summarized in Table 1.

### Case 1

**Figure 1:**
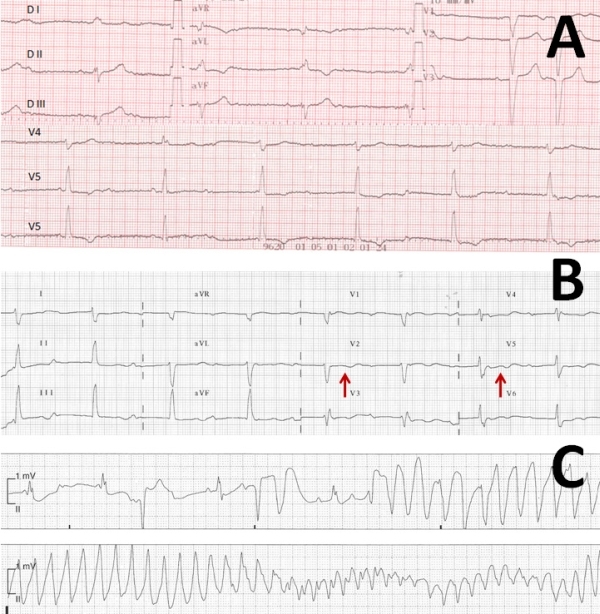
A) 12-lead ECG of Case 1 documented 5 days before the development of TdP showed complete AV block with QT and QTc intevals of 420 msec and 342 ms, respectively. B) ECG on the day of development of torsades demonstrated further QT prolongation and notched T waves (arrows). C) Documented episode of TdP during ECG monitorization in coronary care unit.

An 88-years-old female patient with complaints of fatigue and dizziness was admitted by a primary care physician. Five days later, she had been transferred to our institution for further evaluation of necessity for a permanent PM implantation due to CAVB. Her medical history revealed hypertension. She was on amlodipine for hypertension. Her physical examination revealed mild systolic murmur at the 4th intercostal space. Her heart rate was 50 bpm and blood pressure 120/70 mmHg. Her initial ECG, documented 5 days ago at the time of her first admission, had demonstrated CAVB with QT and QTc intervals of 420 and 342 ms, respectively (Figure 1A). The ECG obtained at her admission to our institution revealed CAVB with further prolonged QT and QTc intervals of 604 and 540 ms, respectively (Figure 1B). Additionally, prominent notches were noticed on the T waves on the second ECG (Figure 1B, arrows). Her electrolyte levels were in normal ranges. Since her clinical and hemodynamical status were stable, we did not implant a temporary PM immediately. A permanent PM implantation was scheduled for the next morning. During the follow-up in the coronary care unit, ECG monitoring revealed episodes of TdP (Figure 1C). One of TdP's degenerated to ventricular fibrillation. She was defibrillated immediately and a temporary PM was implanted. Temporary ventricular pacing at a rate of 90 bpm decreased QT interval to 400 ms and no recurrences of TdP were recorded later on. A DDD-R permanent PM was implanted on the next day.

### Case 2

**Figure 2:**
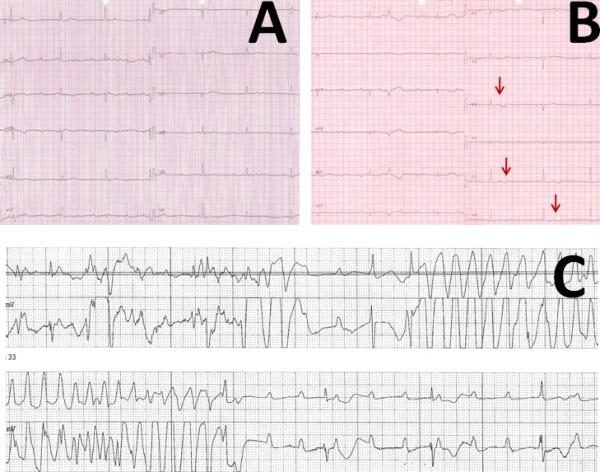
A) 12-lead ECG of Case 2 on the admission day demonstrated complete AV block with QT and QTc intervals of 510 and 406 ms, respectively. B) ECG obtained on the day of development of torsades demonstrated further QT prolongation and notched T waves (arrows). C) Documented episode of TdP during ECG monitorization in coronary care unit.

The second patient was an 85-years-old female with complaints of dizziness, fatigue and presyncope. Her medical history was remarkable with coronary artery disease, hypertension, diabetes mellitus and Alzheimer's diease. She was on multiple drug therapy, including metformin, ramipril, amlodipine, atorvastatin, doxazosin, clopidogrel, memantine, paroxetine and mianserine for these diseases. On the physical examination, her heart rate and blood pressure were 38 bpm and 160/60 mmHg, respectively. ECG revealed CAVB with a heart rate of 38 bpm and narrow QRS. Additionally, QT and QTc intervals on admission were 510 and 406 ms, respectively. Her electrolyte levels were within normal ranges. Her all medications having potential to prolong QT interval were stopped. Since the patient was hemodynamically and clinically stable, she was followed up in the coronary care unit and was scheduled for a permanent PM implantation on the next day. 12-lead ECG obtained on the next day demonstrated further prolongation of the QT and QTc intervals. QT and QTc intervals were 615 and 471 ms, respectively. Furthermore, T wave morphology was different compared to the first ECG. T waves were notched on the second ECG (Figure 2B, arrows). While waiting for PM implantation, episodes of TdP were recorded. Transvenous temporary PM was immediately implanted. Institution of physiological rate via temporary ventricular pacing decreased QT interval to 410 ms and prevented from recurrences of torsades.

### Case 3

**Figure 3:**
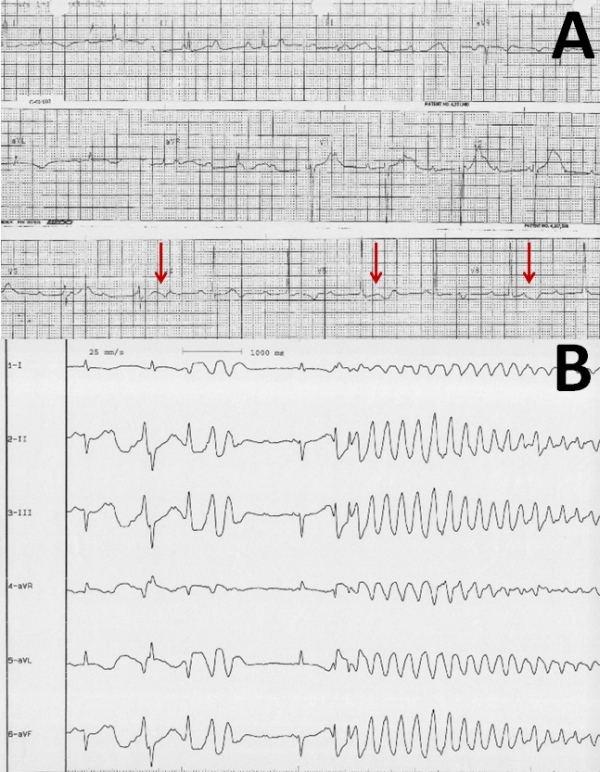
A) 12-lead ECG of Case 3 three weeks after the diagnosis of complete AV block when her complaint of syncope begun, showed complete AV block with QT and QTc intervals of 600 and 531 ms, respectively. B) Documented episode of TdP during ECG monitorization in catheterization laboratory.

The third patient was a 82-years-old female. She was admitted to our department with syncope. She was on lisinopril+hydrochlorothiazide, aspirin and isosorbide mononitrate for hypertension and coronary artery disease. Three weeks earlier, while she was having complaints of dizziness and fatigue, she was diagnosed with CAVB. At that time, she had refused permanent PM implantation. Her physical examination was unremarkable, except bradycardia with a rate of 47 bpm. The ECG demonstrated CAVB with QT and QTc intervals of 600 and 531 ms, respectively (Figure 3A). There were prominent notches on the T waves, especially in the leads V3-6 (Figure 3A, arrows). Her electrolyte levels were within normal ranges. Because of her new emerged complaint of syncope, she gave consent for permanent PM implantation. Before permanent PM implantation, during her stay in the coronary care unit, episodes of TdP were noticed on ECG monitor (Figure 3B). After achieving the physiological ventricular rate via temporary PM, episodes of torsades disappeared.

All of the patients with TdP were females whereas 52% of all patients with CAVB were males (p< 0.05). Mean age of patients with TdP was significantly greater than the mean age of the patients without TdP (p< 0.05, 85±3 vs 78±7.6, respectively). The most prominent common clinical feature of the patients with TdP was the extended exposure to bradycardia, because of the delay in PM implantation. In patients with TdP, the elapsed times from the beginning of block to PM implantation were 48 hours, 5 days and 20 days. In our archives, bradycardia exposure time could be determined in only 49 patients without TdP. Among them, just 10 patients were exposed to bradycardia for over 48 hours, whereas all 3 patients with TdP were exposed to bradycardia over 48 hours (p< 0.05). As expected, all the patients with TdP had prolonged QT and QTc intervals on ECG. Additionally, in two patients with TdP, we demonstrated that QT and QTc prolongation increases as the duration of bradycardia extends. Furthermore all the patients with TdP had notched T waves in the ECG on the occurrence day of TdP.

## Discussion

In this retrospective case-control study, it was found that female gender, advanced age and extended exposure to bradycardia were foretelling TdP in CAVB patients. Complete AV block causes bradycardic ventricular remodelling associated with electrophysiological abnormalities reminiscent of those found in congenital and acquired LQTS's [2,4]. These include repolarization delay manifesting electrocardiographically as QT interval prolongation, and predisposition to the triggered form of polymorphic ventricular tachycardia known as TdP [2]. QT interval is measured from the onset of QRS complex to the termination of T wave [5]. The longest QT intervals are generally measured in precordial leads V3-V4. The QT interval normally shortens with tachycardia and extends with bradycardia. Therefore, a rate corrected QT (QTc) interval should be calculated. Bazett's formula, in which the longest QT interval divided by the square root of the RR interval is the gold standart for QTc calculation [5]0. QTc intervals < 440 ms are clearly normal. In all of our three cases, QTc intervals were abnormally longer than 440 ms on the occurrence day of TdP (Table 2).

Additionally, CAVB may cause a hyperadrenergic state and ventricular ectopy, which could result in a long-short phenomenon and TdP, when timed appropriately.[6,7] This could potentially happen in a bradycardic state without significant prolongation of QT interval and T-wave abnormalities.

According to the ACC/AHA guidelines, pacemaker implantation is a class I indication for CAVB, associated with symptomatic bradycardia (including heart failure) or ventricular arrhythmias presumed to be due to AV block. [8] However, permanent PM implantation is a class IIa indication for persistent third-degree AV block with an escape rate greater than 40 bpm in asymptomatic adult patients without cardiomegaly. In the current AHA/ACC guidelines, QT prolongation is not referred as an indication for PM implantation in CAVB. It is known that bradycardia prolongs the QT interval. [9] Studies show that prolongation of ventricular repolarization which is manifested as QT prolongation, is a prerequisite for TdP. [2] Furthermore, some patients with bradycardia have an increased risk for TdP. [9] In their retrospective case-control study, Topilski and colleagues demonstrated that some patients have relatively higher risk for TdP in case of bradycardia. [2] They have demonstrated that female patients with QT interval>510 ms, QTc interval>400 ms, or Tpeak-Tend>85 ms, especially if these patients also have type 2 LQTS like notched T waves (arrows in Figure 1B, 2B, and 3A), are at sufficiently high risk for developing TdP. In accordance with these findings, all of our three patients were females and had QT and QTc intervals longer than 510 and 400 ms, respectively (Table 1). T waves of the cases were also notched and Tpeak-Tend were longer than 85 ms.

Increased susceptibility to QT prolongation and related TdP is a well known feature of female gender. Kawasaki et al. have also demonstrated that female patients with CAVB experience more often TdP than male patients. [9]

Patients with TdP were older than patients without TdP. Our three cases were older than 80 years. Although previously not reported, advanced age may be a factor for the development of TdP in CAVB patients. The number of our cases is very low when compared with the number of Topilski's study. They have reported 30 bradicardic patients complicated by TdP. They have showed that advanced age is not a predictor for the development of TdP. Our findings may be resulting from statistical error due to the low number of cases. These data show that all results need to be verified by studies performed in higher series of patients.

One of the most prominent common clinical characteristics of our cases was the delay in achieving the physiological ventricular rate. Vos et al showed that torsades inducibility is increased as the bradycardia duration is prolonged. [10] In two of our patients, urgent temporary PM implantation was not performed at the admission due to the absence of hemodynamic instability. Because of our laboratory schedule, permanent PM implantation was planned for the next date available. Supporting the findings of Vos et al., time dependent QT prolongation and QT interval morphological changes were documented in two patients. Chronic CAVB affects ventricular myocyte repolarizing currents of IKr and IKs via K+ channel downregulation and thereby leads to cellular action potential prolongation and early after-depolarizations, considered to be a trigger for TdP. [11] K+ channel downregulation may be augmented with time and increased K+ channel downregulation may be the reason of TdP in patients with delayed pacemaker implantation. Our understanding is that bradycardia induced QT prolongation occurs more often in elderly female patients. Especially, if the duration of bradycardia increases, QT interval prolongs much more and TdP susceptibility increases.

The other most encountered reason for TdP is drug-induced long QT syndrome. TdP is most commonly caused by antiarrhytmic medications. [12] Additionally, dose-dependent QT prolongation has been observed with psychiatric drugs. [13] In the second case, polytherapy with known QT prolonging drugs of paroxetine and mianserin may be the other contributing factors for the development of TdP in addition to CAVB. [14, 15] 

Animal studies have demonstrated that the remodelling process can be aborted entirely by prompt institution and maintenance of physiologic-rate via ventricular pacing. [16] In our cases, dramatic QT interval normalization just after the institution of physiologic ventricular rate via temporary ventricular pacing was also noted. It is recommended that pacemakers implanted for bradycardia-induced TdP should be programmed for a pacing rate of =80 bpm, until the QT interval shortens. [2]

The major limitation of this study is the low number of patients with TdP. Documenting and reporting episodes of TdP is not easy, because the frequency of TdP is not so high to report more patients.

In summary, CAVB may cause QT prolongation predisposing TdP. Delay in PM implantation for the treatment of CAVB may cause further QT prolongation, and thereby TdP. TdP risk is more increased in female patients aged 80 years or older and having abnormally prolonged QT interval and notched T waves on ECG. Monitoring of QT and QTc intervals and also T wave notching in CAVB may be lifesaving. PM implantation should not be delayed in this group of patients because of the increased risk of TdP.

## Figures and Tables

**Table 1:**
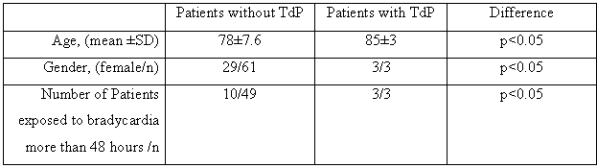
The Differences Between Patients With and Without TdP

TdP: torsades de point.

**Table 2:**
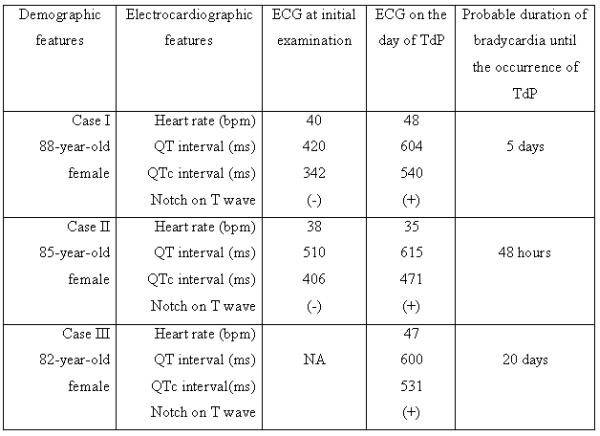
Electrocardiographic Features of the Patients with Torsades de Pointes

NA: not available.
